# Efficacy, safety and biomarker analysis of durvalumab in patients with mismatch-repair deficient or microsatellite instability-high solid tumours

**DOI:** 10.1186/s12885-023-10663-2

**Published:** 2023-03-04

**Authors:** Birgit S. Geurts, Thomas W. Battaglia, J. Maxime van Berge Henegouwen, Laurien J. Zeverijn, Gijs F. de Wit, Louisa R. Hoes, Hanneke van der Wijngaart, Vincent van der Noort, Paul Roepman, Wendy W. J. de Leng, Anne M. L. Jansen, Frans L. Opdam, Maja J. A. de Jonge, Geert A. Cirkel, Mariette Labots, Ann Hoeben, Emile D. Kerver, Adriaan D. Bins, Frans G.L. Erdkamp, Johan M. van Rooijen, Danny Houtsma, Mathijs P. Hendriks, Jan-Willem B. de Groot, Henk M. W. Verheul, Hans Gelderblom, Emile E. Voest

**Affiliations:** 1 Division of Molecular Oncology & Immunology, Netherlands Cancer Institute, Amsterdam, the Netherlands; 2 Oncode Institute, Utrecht, the Netherlands; 3 Department of Medical Oncology, Leiden University Medical Centre, Leiden, the Netherlands; 4 Department of Medical Oncology, Amsterdam University Medical Centre, location VUMC, Amsterdam, the Netherlands; 5 Department of Biometrics, Netherlands Cancer Institute, Amsterdam, the Netherlands; 6 Hartwig Medical Foundation, Amsterdam, the Netherlands; 7 Department of Pathology, University Medical Cancer Centre Utrecht, Utrecht, the Netherlands; 8 Department of Clinical Pharmacology, the Netherlands Cancer Institute, Amsterdam, the Netherlands; 9 Department of Medical Oncology, Erasmus MC Cancer Institute, Rotterdam, the Netherlands; 10 Department of Medical Oncology, Meander, Amersfoort, the Netherlands; 11 Department of Medical Oncology, Department of Internal Medicine, GROW-School for Oncology and Developmental Biology, Maastricht University Medical Centre, Maastricht, the Netherlands; 12 Department of Medical Oncology, Onze Lieve Vrouwe Gasthuis, Amsterdam, the Netherlands; 13 Department of Medical Oncology, Amsterdam University Medical Centre, location AUMC, Amsterdam, the Netherlands; 14 Department of Medical Oncology, Zuyderland Hospital, Sittard-Geelen, the Netherlands; 15 Department of Medical Oncology, Martini Hospital, Groningen, the Netherlands; 16 Department of Medical Oncology, Haga Hospital, The Hague, the Netherlands; 17 Department of Medical Oncology, Northwest Clinics, Alkmaar, the Netherlands; 18 Department of Medical Oncology, Isala, Zwolle, the Netherlands

**Keywords:** Durvalumab, Immunotherapy, Microsatellite instability, Mismatch repair deficiency, Precision medicine

## Abstract

**Background:**

In this study we aimed to evaluate the efficacy and safety of the PD-L1 inhibitor durvalumab across various mismatch repair deficient (dMMR) or microsatellite instability-high (MSI-H) tumours in the Drug Rediscovery Protocol (DRUP). This is a clinical study in which patients are treated with drugs outside their labeled indication, based on their tumour molecular profile.

**Patients and methods:**

Patients with dMMR/MSI-H solid tumours who had exhausted all standard of care options were eligible. Patients were treated with durvalumab. The primary endpoints were clinical benefit ((CB): objective response (OR) or stable disease ≥16 weeks) and safety. Patients were enrolled using a Simon like 2-stage model, with 8 patients in stage 1, up to 24 patients in stage 2 if at least 1/8 patients had CB in stage 1. At baseline, fresh frozen biopsies were obtained for biomarker analyses.

**Results:**

Twenty-six patients with 10 different cancer types were included. Two patients (2/26, 8%) were considered as non-evaluable for the primary endpoint. CB was observed in 13 patients (13/26, 50%) with an OR in 7 patients (7/26, 27%). The remaining 11 patients (11/26, 42%) had progressive disease. Median progression-free survival and median overall survival were 5 months (95% CI, 2-not reached) and 14 months (95% CI, 5-not reached), respectively. No unexpected toxicity was observed. We found a significantly higher structural variant (SV) burden in patients without CB. Additionally, we observed a significant enrichment of *JAK1* frameshift mutations and a significantly lower IFN-γ expression in patients without CB.

**Conclusion:**

Durvalumab was generally well-tolerated and provided durable responses in pre-treated patients with dMMR/MSI-H solid tumours. High SV burden, *JAK1* frameshift mutations and low IFN-γ expression were associated with a lack of CB; this provides a rationale for larger studies to validate these findings.

**Trial registration:**

Clinical trial registration: NCT02925234. First registration date: 05/10/2016.

**Supplementary Information:**

The online version contains supplementary material available at 10.1186/s12885-023-10663-2.

## Background

Mismatch repair deficient (dMMR) or microsatellite instability-high (MSI-H) tumours comprise 2 to 4% of all diagnosed cancers and are most commonly observed in colorectal, endometrial and gastric adenocarcinomas [1–3]. dMMR/MSI-H tumours have a unique genetic signature caused by germline or acquired deficiency of one of the four major mismatch repair genes*, MLH1*, *MSH2*, *MSH6* and *PMS2* [4, 5]. The protein functions are achieved by heterodimers, MLH1 being the PMS2 partner and MSH2 being the MSH6 partner [6]. Deficiencies in the major mismatch repair genes lead to insertions and deletions (indels) in highly repetitive DNA sequences, termed microsatellites, resulting in a higher degree of microsatellite instability (MSI) [2, 5, 7, 8]. As a consequence, these tumours have an exceptionally high number of somatic mutations, especially frameshift indels, generating a high burden of neoantigens [2, 9–11]. Therefore, dMMR/MSI-H tumours are considered to be highly immunogenic, rendering them more sensitive to programmed cell death 1 (PD-1) and programmed cell death ligand 1 (PD-L1) inhibitors [2, 9].

The inhibitory ligand PD-L1 is frequently upregulated in tumours cells, which results in the exhaustion of cytotoxic T cells by binding to PD-1 and contributes to tumour immune escape. This can be reversed by PD-1 or PD-L1 immune checkpoint inhibitors (ICI), thereby restoring anti-tumour immunity [12, 13]. Sensitivity to PD-1 inhibitors, such as nivolumab and pembrolizumab, has been frequently observed across various dMMR/MSI-H tumours. The CheckMate 142 study showed that nivolumab provided durable responses in pre-treated patients with dMMR/MSI-H metastatic colorectal cancer (CRC) and observed an objective response rate (ORR) of 31.1% (95% confidence interval (CI), 20.8–42.9%) [14]. Moreover, the KEYNOTE-158 study observed similar results with pembrolizumab in pre-treated patients with non-CRC dMMR/MSI-H tumours and showed an ORR of 34.3% (95% CI, 28.3–40.8%) [2]. Efficacy of anti-PD1 has also been investigated in first-line metastatic setting. The KEYNOTE-177 study showed that pembrolizumab improved progression free survival (PFS) as first-line therapy in metastatic dMMR/MSI CRC compared to standard of care chemotherapy [15, 16]. Results from these studies have led to several approvals by the Food and Drug Administration, including the first tumour-agnostic authorization for pembrolizumab in unresectable or metastatic dMMR/MSI-H tumours that have progressed after prior standard treatment and lack satisfactory alternative treatment options [17].

Efficacy of PD-L1 inhibitors however, has mainly been described in a subset of dMMR/MSI-H tumours. The PHAEDRA study showed promising activity of durvalumab in 35 patients with advanced dMMR/MSI-H endometrial cancer and found an ORR of 47% (95% CI, 32–63%), consistent with previous trials evaluating the efficacy of anti-PD1 in dMMR/MSI-H tumours [2, 18–20]. In 30 patients with metastatic dMMR/MSI-H CRC, durvalumab also showed encouraging activity equivalent to that of PD-1 inhibitors with an ORR varying between 27% (95% CI, 0.6–61%) and 42.4% (95% CI, 25.5–60.8%) [21, 22]. Furthermore, the SAMCO-PRODIGE 54 trial showed that the PD-L1 inhibitor avelumab was superior to chemotherapy with respect to PFS with a 12-month PFS of 19% and 31% in the control and avelumab group, respectively [23]. However, evidence regarding efficacy of PD-L1 inhibitors in other dMMR/MSI-H solid tumours remains limited. Therefore, we evaluated the efficacy and safety of durvalumab, a human immunoglobulin G1 kappa monoclonal antibody with high affinity and selectivity against PD-L1 [24], across various dMMR/MSI-H solid tumours in the Drug Rediscovery Protocol (DRUP). DRUP is an ongoing prospective, multicentre, non-randomized clinical trial in which cancer patients, who have exhausted all standard of care options, are treated with approved targeted- or immunotherapies outside their registered indication, based on their tumour molecular profile [25]. DRUP aims to facilitate patient access to commercially available anti-cancer drugs and to describe efficacy and safety data of these drugs when used outside their registered indication. Furthermore, DRUP also creates a unique opportunity to explore determinants of (non-) response by performing extensive biomarker analyses on mandatory fresh frozen tumour biopsies.

## Methods

### Study design

DRUP is an ongoing prospective, multicentre, non-randomized clinical umbrella and basket trial in which patients with metastatic or advanced solid tumours, non-Hodgkin lymphoma or multiple myeloma, without standard of care options, are treated based on their tumour molecular profile with targeted- or immunotherapy outside their registered indication. Patients are enrolled in parallel cohorts, each defined by one tumour type, one molecular variant and one study treatment. For selected biomarkers, such as dMMR/MSI-H, the protocol allows for tumour-agnostic cohorts [25].

DRUP is registered with ClinicalTrials.gov, number NCT02925234. DRUP was approved by the independent ethics committee and by the institutional review boards in every participating hospital. The study is conducted in accordance with Good Clinical Practice guidelines and the Declaration of Helsinki’s ethical principles for medical research. Written informed consent was obtained from all study subjects [25].

### Study population

Eligible patients were adults aged ≥ 18 years with advanced solid tumours who had exhausted all standard of care options. Patients were eligible if routine molecular testing demonstrated dMMR by loss of staining of one of the mismatch repair proteins MLH1, MSH2, MSH6 and PMS2 by immunohistochemistry (IHC) or MSI-H by either polymerase chain reaction (PCR), panel-based next generation sequencing (NGS) or whole genome sequencing (WGS). Patients had measurable disease according to the Response Evaluation Criteria in Solid Tumours version 1.1 (RECIST v1.1) [26], or according to Response Assessment in Neuro-Oncology (RANO) [27] criteria, an acceptable organ function and an Eastern Cooperative Oncology Group performance status of 0–1. Patients were considered evaluable if response was radiologically or clinically evaluable, if they received at least one treatment administration and if they were on study for at least one treatment cycle. Non-evaluable patients were replaced and excluded from biomarker analyses.

### Treatment assessment and evaluation

Patients were treated with monotherapy durvalumab (1500 mg intravenously every 4 weeks) until disease progression or unmanageable toxicity. Treatment beyond progression was not permitted in the protocol. Radiological imaging for tumour response assessment was performed at baseline and every 8 weeks (2 cycles) after treatment initiation.

Safety was measured by the frequency of grade ≥ 3 treatment related adverse events (AEs) occurring up to 30 days after the last administration of study drug. All AEs were graded according to the Common Terminology Criteria for Adverse Events (CTCAE) v4.03.

The primary endpoints of this study, as previously described [25, 28, 29], were clinical benefit (CB) and safety. CB was defined by confirmed complete or partial response (CR; PR) or stable disease (SD) for at least 16 weeks, according to RECIST 1.1. or RANO criteria and measured at least two times, at least 28 days apart in a particular cohort. Safety was defined as grade ≥ 3 treatment-related AEs. Secondary endpoints included PFS and overall survival (OS). Biomarker analyses on pre-treatment biopsies formed an exploratory endpoint.

### Pre-treatment biopsies and biomarker analysis

According to protocol, it was mandatory to obtain a fresh frozen tumour biopsy before start of treatment. Exceptions were made for patients with primary brain tumours. DNA was isolated from biopsies and all biopsies were analysed for WGS (the Hartwig Medical Foundation, Amsterdam, The Netherlands) on the Illumina Novaseq (2 × 151) platform, together with a matched 10-ml blood sample to determine germline DNA of a patient. If the tumour-cell percentage was ≥20% and the DNA yield was ≥300 ng, WGS and RNA sequencing were performed. Hartwig Medical Foundation provides high quality sequencing data as Priestley et al. [30] described with a median average depth of 106x (tumour) and 38x (blood). WGS analysis was performed as previously described [30, 31] whereby somatic single nucleotide variants and indels were called using SAGE, and purity and ploidy estimations, structural variant (SV) and copy number analysis were performed using HMF’s in-house tools GRIDSS, PURPLE and LINX. Tumour mutation burden (TMB) per megabase (Mb), tumour mutational load (ML) and microsatellite-instable indels (msIndels) per Mb were calculated by computing the number of total somatic mutations, the number of missense mutations and number of frameshifts in microsatellite regions, respectively. SV burden was defined as the sum of total number of non-inferred and non-single passing SVs per sample.

Total RNA was extracted using the QIAGEN QIAsymphony RNA kit. Samples with approximately 100 ng total RNA were prepared with KAPA RNA Hyper + RiboErase HMR and RNA libraries were paired end sequenced on the Illumina NextSeq550 platform (2x75bp) or Illumina NovaSeq6000 platform (2x150bp). Gene expression was quantified using Salmon (v1.60) and IFN-gamma (IFN-γ) expression was computed using gene sets as previously described [32].

### Statistical analysis

In DRUP, as previously described [25, 28, 29], cohorts are monitored using a Simon-like two-stage “admissible” monitoring plan to identify cohorts with evidence of activity [33]. If no CB is observed in any of the first enrolled 8 patients in the cohort, the cohort will be closed. Otherwise, an additional 16 patients will be included in the cohort. Four or fewer patients with CB would suggest a lack of activity, whereas five or more patients with CB will suggest that further investigation of the drug in the tumour/variant is warranted. The null hypothesis and alternative hypothesis to be tested are defined as clinical benefit rate (CBR) of 10% versus ≥ 30%. This design has 85% power to reject the null hypothesis of a CBR of 10% when the true CBR is 30%, with a one-sided alpha error rate of 7.8% [29]. Cohorts with a response rate of ≥ 30% are considered potentially successful and may proceed to stage III in DRUP to validate and confirm results earlier found [34].

All statistical analyses were performed using R version 4.0.3. Patient characteristics, AEs and tumour responses were summarized using descriptive statistics. Kaplan-Meier methods were used to estimate PFS (from start treatment to progression or death from any cause and censoring patients alive without progression) and OS (calculated from the first day of treatment administration to the date of death from any cause, censoring patients who were alive at last follow-up). Duration of response (DoR) was calculated from the first date response was measured until disease progression. Differences in CB between different groups of patients were analysed using the Fisher’s exact test. Differences of genomic features in patients with CB and without CB were compared using the Mann-Whitney U test.

## Results

### Accrual and patient characteristics

From January 2019 through April 2020, a total of 47 patients with histologically confirmed dMMR/MSI-H solid tumours who had exhausted all standard of care options, were submitted to the central study team for evaluation for potential study participation in the cohort “Durvalumab for dMMR/MSI-H tumours”. Forty-three patients were approved by the central study team to be screened for treatment with durvalumab, but 17 patients dropped out after allocation. Of those, four patients underwent alternative treatment options and four patients preferred not to undergo study treatment. The remaining nine patients did not meet the inclusion criteria, mainly due to rapid clinical deterioration (*n* = 6, 67%) (Supplemental Fig. S1). Twenty-six patients with ten different tumour types were considered eligible and started study treatment, of which the majority (*n* = 8, 31%) had CRC. Twenty-four patients were evaluable for the primary endpoint. Two patients were not evaluable for the primary endpoint according to our protocol definition on treatment evaluability (one patient had rapid clinical deterioration and one patient had disease progression confirmed by radiological imaging, both within the first treatment cycle). Baseline characteristics are presented in Table 1.

**Table 1:** Baseline characteristics of the 26 patients enrolled

Characteristics	Number (%) of patients	
**Age (approximately at consent)**		
Median (range)	64.5	(34–81 years)
**Gender**		
Male	14	54%
Female	12	46%
**ECOG performance status**		
ECOG 0	8	31%
ECOG 1	18	69%
**Primary tumour types**		
Colorectal cancer	8	31%
Endometrial cancer	3	12%
Small intestine cancer	3	12%
Stomach cancer	3	12%
Bile duct cancer	3	12%
Breast cancer	2	8%
Pancreatic cancer	1	4%
Prostate cancer	1	4%
Neuroendocrine cancer	1	4%
Glioblastoma	1	4%
**Number of prior systemic therapy lines**^**a**^		
No previous lines	1	4%
1 previous line	10	38%
2 previous lines	7	27%
≥ 3 previous lines	8	31%
**Lynch syndrome**	8	33%

Percentages may not equal 100% due to rounding

*Abbreviations: ECOG* Eastern Cooperative Oncology Group

^a^Number of prior systemic therapy lines is the sum or prior lines of hormonal therapy, chemotherapy and targeted-therapy

### Clinical benefit and safety

Thirteen out of 26 patients (50%, 95% CI, 30–70%) had CB upon treatment with durvalumab. An objective response (OR) was observed in 7 out of 26 patients (27%, 95% CI, 12–48%); three patients achieved a CR (3/26, 12%) and four patients achieved a PR (4/26, 15%; Supplemental Table S1). The remaining 11 evaluable patients (11/26, 42%) had progressive disease. CB was more frequently observed in patients with CRC vs non-CRC tumours (85% vs. 41%); however, this difference was not statistically significant (*p* = 0.08).

**Fig. 1:**
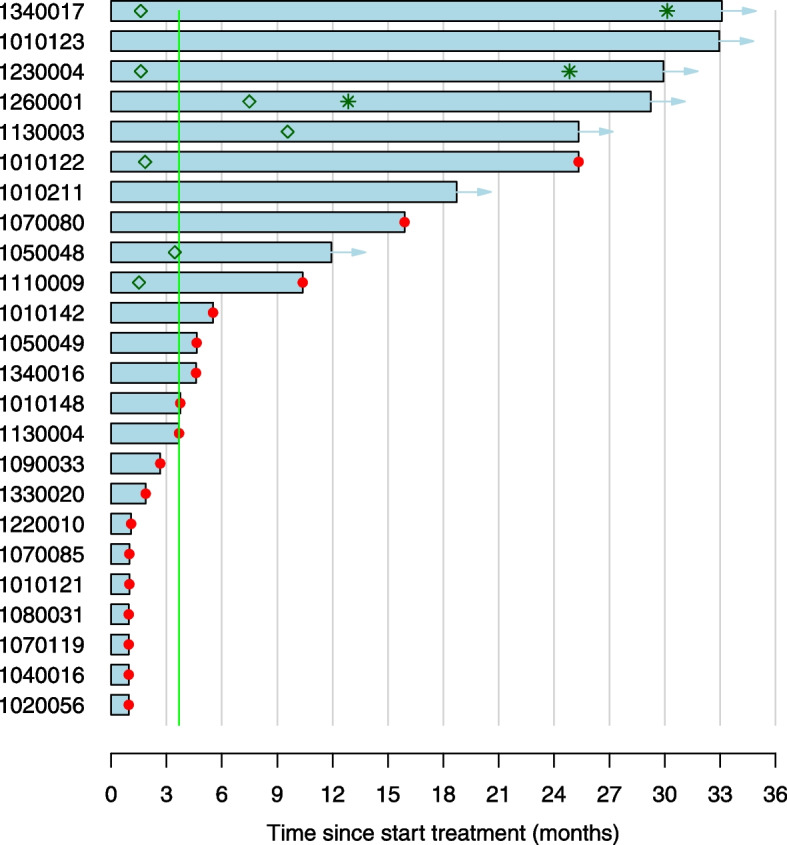
Treatment efficacy of durvalumab. Swimmer plot of the time on treatment (in months) for each evaluable patient (*n* = 24). Patients marked with an arrow were still on study (as per December 16th, 2021). The red dot marks treatment discontinuation. The diamond-shape marks partial responses (PR) and the asterisk marks complete responses (CR)

**Fig. 2:**
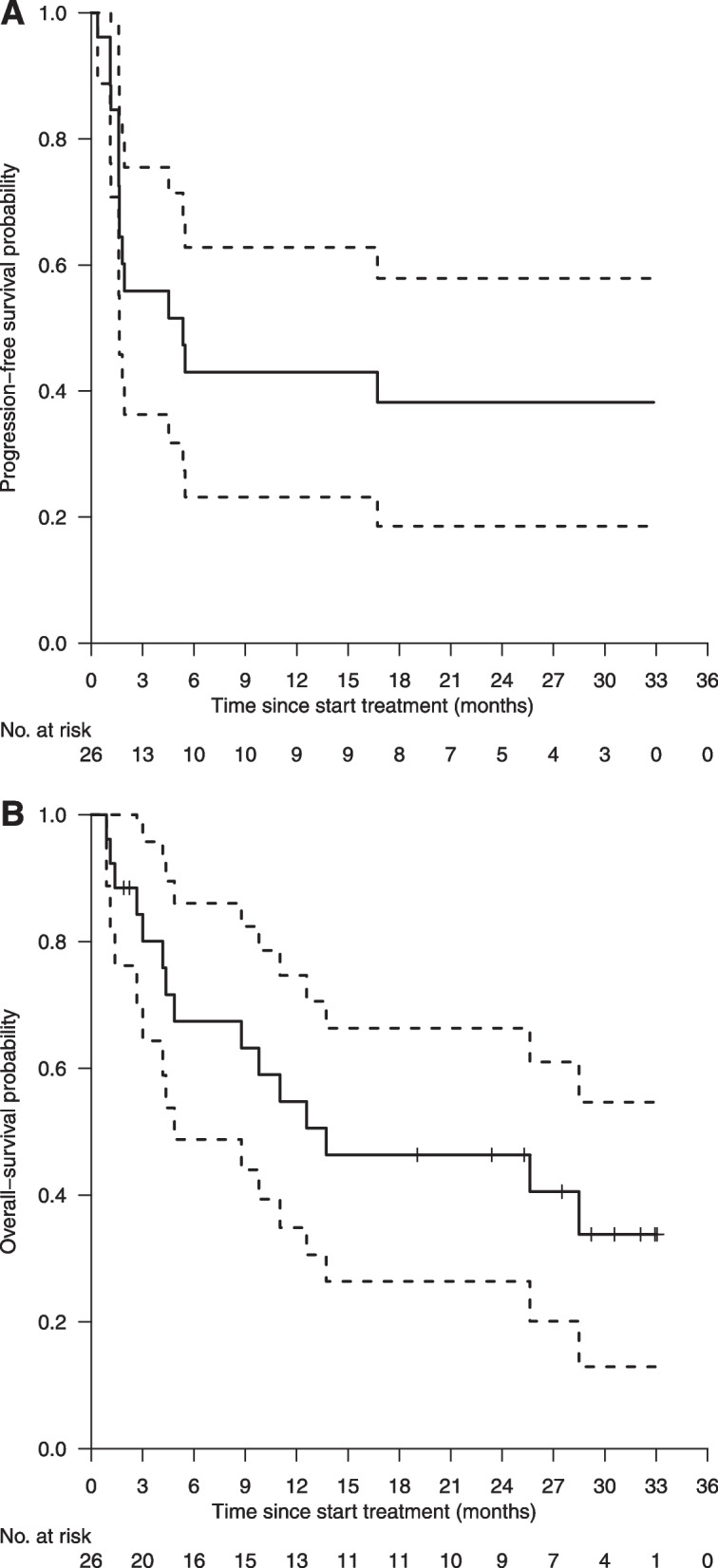
**A** Progression Free Survival curve. **B** Overall Survival curve. Legend: Progression free survival (PFS) and overall survival (OS) of each enrolled patient (*n* = 26) in the cohort “Durvalumab for dMMR/MSI-H tumours”. Kaplan-Meier curves for estimated PFS (**A**) and OS (**B**), with 95% Confidence Intervals (dashed lines)

At data cut-off (16th of December, 2021), after a median follow-up of 29 months (95% CI, 23–32 months), the median duration of response was not yet reached (95% CI, 15–NA months) and seven patients were still on study. The median time on treatment was 4.2 months (95% CI, 1.1–15.9 months; Fig. 1). Median PFS and OS were 5 months (95% CI, 2–NA months; Fig. 2A) and 14 months (95% CI, 5–NA months; Fig. 2B), respectively.

Overall, durvalumab was well-tolerated. Grade ≥ 3 AEs occurred in 15 patients (15/26, 58%) and treatment-related grade ≥ 3 AEs occurred in 5 patients (5/26, 19%) (Table 2). In one patient treatment was discontinued due to development of a grade 3 contained gastric perforation at the tumour site, which was considered to be possibly treatment-related. This patient went off study and was lost to follow-up. No grade ≥ 3 immune related AEs, grade 5 AEs and serious unexpected AEs occurred.

**Table 2:** Adverse events of grade 3 and higher

Adverse event	Grade 3	Grade 4	Grade 5
Acute kidney injury	1		
**AP increased**^**a**^	2		
Anaemia	2		
Anorexia	1		
ASAT increased	1		
Bile duct stenosis	1		
Cholangitis	2		
Chronic kidney disease	1		
**CK increased**^**a**^	1		
**Dyspnoea**^**a**^	1		
Fatigue	1		
**Gastric perforation**^**a**^	1		
Gastro intestinal haemorrhage	1		
Gastrointestinal abdominal pain	2		
**GGT increased**^**a**^	1	1	
Hypertension	1		
Hypophosphatemia	1		
Ileus	2		
**WBCC decreased**^**a**^	1		
Pneumothorax left	1		
**Thromboembolic event**^**a**^	2		
Urinary tract infection	1		

All the adverse events of grade 3 or higher. For each adverse event, the number of patients is displayed in whom it was reported at grade 3, 4 or 5 as the highest grade

*Abbreviations: AP* alkaline phosphatase, *ASAT* aspartate aminotransferase, *CK* creatinine phosphokinase, *GGT*, gamma-glutamyl transpeptidase, *WBCC* white blood cell count

^a^For the adverse events in bold, the relation to the treatment was scored as either ‘possible’, ‘probable’ or ‘definite’

### Baseline biopsies sequencing and MSI classification

Pre-treatment biopsies were obtained in 25 out of 26 enrolled patients. One patient with a primary brain tumour did not undergo a biopsy according to protocol. Biopsies from six patients (6/26, 23%) could not be sequenced due to insufficient tumour purity (< 20%). Therefore, 19 biopsies had sufficient material for WGS sequencing (19/26, 73%) and 14 biopsies (14/19, 74%) were also available for RNA sequencing.

Concordance between MSI classification based on WGS data and IHC analysis was observed in 17 patients (17/19, 89%). Two patients were classified as microsatellite stable (MSS) by WGS. One of these patients with endometrial cancer was enrolled based on IHC indicating loss of MLH1/PMS2 and no methylation of *MLH1*. However, WGS revealed a clear MSS lesion with a msIndel burden of only 0.28 indels/Mb (MSI cut-off: 4 indels/Mb) and low TMB of 3.6 mut/Mb. The other patient was diagnosed with a Lynch associated pancreatic carcinoma and was enrolled because of isolated loss of MSH6 by IHC. WGS indicated MSS with a msIndel burden of 3.31 indels/Mb and high TMB of 21.7 mut/Mb. Both patients (18% of all that had PD) did not experience CB. (Table 3).

**Table 3:** Data on biomarkers

DRUP-ID	Tumour type	Pre-enrolment based on			Baseline WGS					Baseline RNA	BOR
		Confirmed Lynch (germline mutation)	IHC	Molecular analysis	WGS available	MSI	MMR mutations	***B2M***/***JAK1***/***JAK2***/***STAT1***	TMB (mut/Mb)	RNA available	
1010121	Endometrium		MLH1/PMS2 loss		Yes	No			3.6	No	PD
1010122	CRC		MLH1/PMS2 loss		Yes	Yes		*STAT1* p.R70C	193.2	Yes	PR
1010123	Small intestine	Yes^a^	MSH2/MSH6 loss		Yes	Yes	*MSH6*;*MSH2*;*MLH1*	*STAT1* p.D674N	274.7	Yes	SD
1010142	Bile duct		MLH1/PMS2 loss		Yes	Yes	*MLH1*^b^;*MSH6;MLH1*^b^;		48.9	No	SD
1010148	Breast			MSI by WGS (msIndels 45.0)	Yes	Yes	*MSH2*^b^	*JAK1* p.K860Nfs*16;p.P430Rfs*2, JAK2 p.L224*	122.6	Yes	PD
1010167	Endometrium		MLH1/PMS2 loss		Yes	Yes		*JAK1* p.K860Nfs*16	44.8	No	NE
1010211	CRC	Yes (*MSH6)*	MSH6/PMS2 loss		Yes	Yes	*PMS2*;*MSH6*	*B2M* p.Q22*	60.0	Yes	SD
1020053	CRC		MLH1/PMS2 loss	MSI by PCR (pentaplex)	Yes	Yes	*MSH6*;*MSH2*;*MLH1*	*JAK1* p.S771R	366.0	No	NE
1020056	Glioblastoma	Yes (*MSH2*)			No	NA	NA	NA	NA	No	PD
1040016	Small intestine	Yes (*MLH1*)		MSI by PCR (8 marker panel)	Yes	Yes	*MSH6*	*B2M* p.M1?;p.T93Lfs*10, *JAK1* p.K860Nfs*16;p.P430Rfs*2	213.3	No	PD
1050048	Bile duct	Yes^a^	MLH1/PMS2 loss		Yes	Yes	*PMS2*	*B2M* p.S16Afs*27, *JAK2* p.R1113H	127.0	Yes	PR
1050049	Prostate			MSI by WGS (msIndels 35.6)	Yes	Yes	*MSH2*^b^	*JAK1* p.K860Nfs*16;p.P430Rfs*2	65.0	Yes	PD
1070080	CRC		MLH1/PMS2 loss	MSI by PCR (pentaplex)	Yes	Yes	*MSH2*;*PMS2*		145.6	No	SD
1070085	Stomach			MSI by NGS (56 marker panel)	Yes	Yes	*PMS2*		105.8	Yes	PD
1070119	Pancreas	Yes (*MSH6*)	MSH6 loss		Yes	No			21.7	No	PD
1080031	Bile duct		MLH1/PMS2 loss		Yes	Yes			80.0	Yes	PD
1090033	CRC		MLH1/PMS2 loss		No	NA	NA	NA	NA	NA	PD
1110009	Small intestine		MSH2/MSH6 loss	MSI by PCR (pentaplex)	No	NA	NA	NA	NA	NA	PR
1130003	Endometrium	Yes (*MSH2*)			No	NA	NA	NA	NA	NA	PR
1130004	NEC		MLH1 loss		No	NA	NA	NA	NA	NA	SD
1220010	Stomach		MLH1/PMS2 loss		No	NA	NA	NA	NA	NA	PD
1230004	CRC		MLH1/PMS2 loss		Yes	Yes	*MSH6*	*B2M* p.S16Ffs*29;p.S16Afs*27	293.2	Yes	CR
1260001	Stomach		MLH1/PMS2 loss		No	NA	NA	NA	NA	NA	CR
1330020	Breast			MSI by WGS (msIndels 63.5)	Yes	Yes	*MSH6*	*JAK1* p.K860Nfs*16;p.P430Rfs*2, *STAT1* p.A479V	294.0	Yes	PD
1340016	CRC	Yes (*MSH2*)			Yes	Yes	*MSH2*		217.8	Yes	SD
1340017	CRC		MLH1/PMS2 loss		Yes	Yes	*MSH6*; *PMS2*	*B2M* p.M1?;p.L15Ffs*41, *JAK1* p.K860Nfs*16	247.7	Yes	CR

Data on biomarkers of enrolled patients (*n* = 26)

*Abbreviations: BOR* best overall response, *CR* complete response, *CRC* colorectal cancer, *IHC *immunohistochemistry, *MMR *mismatch repair, *Mut/Mb* mutations per megabase, *MSI* microsatellite instability, *msIndels* microsatellite-instable indels, *NA* not applicable, *NE* non-evaluable, *NEC* neuroendocrine carcinoma, *NGS* next generation sequencing, *PCR* polymerase chain reaction, *PD* progressive disease, *PR* partial response, *SD* stable disease, *TMB* tumour mutation burden, *WGS* whole genome sequencing

^a^Germline mutation not known, ^b^bi-allelic loss of heterozygosity of that gene

MSS samples and samples of patients considered as non-evaluable were excluded from the further biomarker analyses. Thus, 15 samples and 12 samples were included in the genomic and transcriptomic analysis, respectively (Table 3).

### Genomic-derived biomarkers of ICI response

**Fig. 3:**
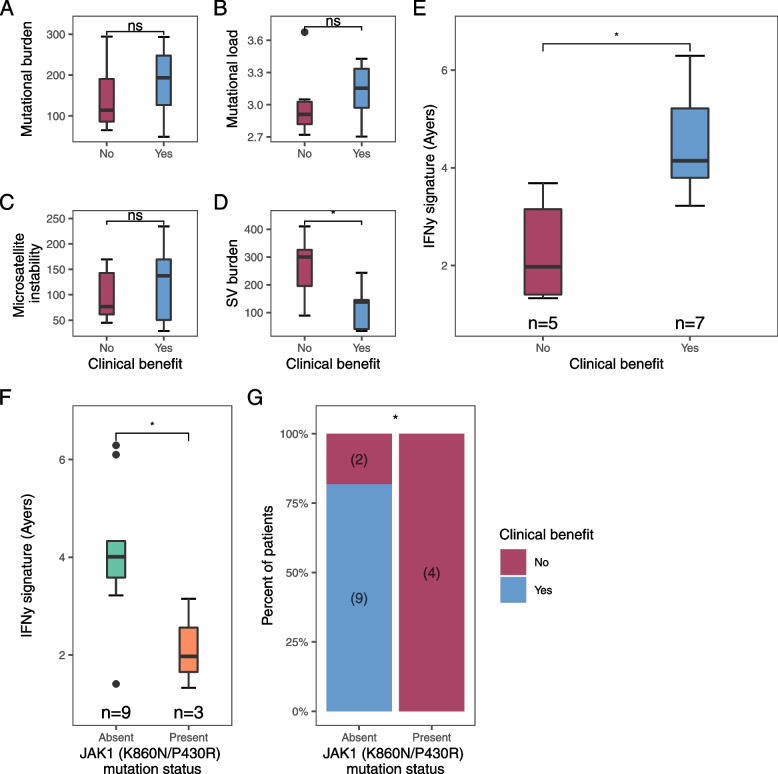
Comparison of genomic features. **A** Tumour mutation burden per megabase, **B** Tumour mutational load (log10), **C** microsatellite-instable indels per megabase, **D** structural variant burden, **E** expression score of IFN-gamma (IFN-γ) in patients experiencing clinical benefit (blue) or no clinical benefit (red), **F** expression score of IFN-γ in patients with two *JAK1* (K860N/P430R) frameshift mutations (orange) or not (green) and **G** proportion of patients with two *JAK1* (K860N/P430R) frameshift mutations or not, by clinical benefit. The box plot shows the median, first and third quartiles, whiskers extend to 1.5 times the interquartile range, and outlying points are plotted individually and two-sided. In **A-F** a two-sided Mann-Whitney U test was used. In **G** a Fisher’s Exact test was used *p < 0.05; ns, not significant

Given previous reports on TMB as an independent biomarker of response in dMMR/MSI-H CRC [35], we next compared TMB of patients with CB and without CB. Generally, we found that patients experiencing CB had a higher TMB than patients without CB (median TMB 193 mut/Mb vs. 114 mut/Mb), but this difference was not statistically significant (*p* = 0.61; Fig. 3A). Tumours with a high ML and/or high msIndel burden harbour an expanded neoantigen repertoire, making them more immunogenic and responsive to ICI [2, 10, 11, 36]. Therefore, we analysed patients by ML and msIndel burden, but we did not observe any statistically significant differences by CB (*p* = 0.22, *p* = 0.61; Fig. 3B**/**C). Patients with CB and without CB had a median ML of 1424 and median msIndel burden of 137 indels/Mb compared with a median ML of 820 and median msIndel burden of 77 indels/Mb, respectively. SVs may result in more foreign neoantigens than neoantigens derived by single mutations or small insertions and deletions [37] and we therefore lastly evaluated SV burden. Interestingly, we observed that patients without CB had a significantly higher SV burden compared to patients with CB (median SV burden 300 vs. 138, *p* = 0.026; Fig. 3D).

### Association between IFN-γ signalling pathway and CB

Anti-tumour immune responses require adequate antigen presentation, which is coordinated by several genes, including *B2M* [38, 39]. However, in our cohort, *B2M* mutations were more often observed in patients with CB (4/5, 80%; Table 3) compared to patients without CB (1/5, 20%; Table 3), but this difference was not statistically significant (*p* = 0.58; Table 3).

Antigen presentation is also mediated by the IFN-γ signalling pathway, which is a critical driver of PD-L1 expression in tumour cells and therefore plays an important role in the efficacy of ICI [32, 38, 39]. We therefore examined mRNA expression of IFN-γ in patients with and without CB and observed that IFN-γ expression was significantly lower in patients without CB (*p* = 0.01; Fig. 3E**)**. The IFN-γ signalling pathway can be modulated by several genes, including *JAK1, JAK2* and *STAT1* [38–40]. Interestingly, we observed the presence of *JAK1* mutations in five patients (5/15, 33%; Table 3), which is comparable to the frequency of *JAK1* mutations among dMMR/MSI-H tumours in The Cancer Genome Atlas (TCGA) dataset (91/400, 23%) [41]. However, within this group, we observed the presence of two concurrent frameshift *JAK1* mutations (K860N/P430R), in four patients (4/5, 80%; Table 3), which is a higher prevalence than found in the TCGA dataset (5/91, 5%) [41]. We found the presence of the two *JAK1* frameshift mutations to be significantly enriched in patients that did not experience CB (*p* = 0.011; Fig. 3G). Furthermore, we found that the presence of the two *JAK1* frameshift mutations was significantly associated with a lower IFN-γ expression compared to tumours without those *JAK1* frameshift mutations (*p* = 0.036; Fig. 3F). Additionally, we did not find any statistically significant enrichment of mutations in *STAT1* and *JAK2* in patients with or without CB (Table 3).

## Discussion

The PD-L1 inhibitor durvalumab provided durable responses in previously treated patients with advanced dMMR/MSI-H solid tumours, with 13 patients (50%) experiencing CB, including 7 patients (27%) with an OR. These findings are in line with previously reported response rates to ICI in pre-treated dMMR/MSI-H tumours [2, 21].

Baseline WGS was successfully performed on 73% of the obtained biopsies. This is consistent with the overall WGS success rate within DRUP [25] and within the Dutch CPCT-02 study [30]. Interestingly, in two patients there was no concordance between WGS and IHC analysis. Both patients did not experience CB, possibly explained by the MSS status. Potential explanations for the discrepancy between IHC and WGS in the patient with somatic endometrial cancer can be misinterpretation of IHC by the pathologist [6] or tumour heterogeneity. Although dMMR is an early event in carcinogenesis, tumour heterogeneity in dMMR (endometrial) tumours has previously been reported [6, 42, 43]. The discrepancy in the patient with Lynch associated pancreatic cancer may be explained by the isolated loss of MSH6, since it has been shown that isolated loss of MSH6 does not always result in complete loss of mismatch repair function [6], which possibly explains why the tumour did not reach the cut-off of a msIndel burden of 4.0 indels/Mb [31]. These data highlight the importance of optimal molecular diagnostics. Additional studies are essential to determine the accuracy of currently used routine tests for dMMR/MSI-H in a pan-cancer setting.

Next, we observed that ML, TMB and msIndels were generally higher in patients with CB than patients without CB, which is line in with previous literature [35, 44, 45]. However, these differences were not significant in this patient cohort, possibly due to small sample size or the inherent differences between tumours depending on subtype. Interestingly, we found that higher SV burden was statistically significantly associated with no CB, which is consistent with previously reported data in melanoma patients treated with ICI [46]. However, research into the role of SVs is limited due to difficulties in detection [47] and therefore their role in resistance to ICI is not entirely clear. Our finding suggests that dMMR/MSI-H tumours with high SV burden are less sensitive to ICI, which mechanistically may be due to the formation of resistance mechanisms generated by structural changes. Further research is required to confirm this observation in order to better understand the possible role of SVs as a potential biomarker in dMMR/MSI-H tumours.

We also explored IFN-γ expression and genes associated with the IFN-γ signalling pathway, as it has been shown that this pathway plays a crucial role in efficacy of ICI [32, 38, 39]. As expected, based on previous literature, we found that patients without CB had significantly lower IFN-γ expression than patients with CB [32]. Furthermore, we observed a significant enrichment of *JAK1* frameshift mutations in patients without CB. These *JAK1* frameshift mutations have previously been described as recurrent mutations and non-functional mutations, especially in dMMR/MSI-H tumours and have been associated with resistance to ICI if complete loss of function occurs [9, 40, 48–51]. The presence of two *JAK1* mutations and the significantly lower IFN-γ expression both suggest complete loss of function of *JAK1*. We therefore considered these *JAK1* frameshift mutations as a possible route for primary resistance mechanism to ICI, which may suggest that patients with dMMR/MSI-H tumours harbouring these *JAK1* frameshift mutations are possibly not good candidates for ICI treatment and should be excluded from this treatment.

Interestingly, we observed the presence of two *JAK1* frameshift in a higher prevalence (4/5, 80%) compared to the TCGA dataset (5/91, 5%). This difference may be influenced by our small sample size or may reflect the fact that the TCGA also includes newly diagnosed dMMR/MSI-H cancers whereas our dataset only consisted of patients with advanced, pre-treated dMMR/MSI-H tumours.

This study has several potential limitations. One limitation is the heterogeneity of this cohort. Ten different tumour types were enrolled, resulting in a heterogeneous study population with large variations in prior treatment regimes. Furthermore, WGS was not in all cases available to confirm MSI status. Additionally, as response evaluations were performed according to RECIST criteria, potential pseudoprogression could not be taken into consideration [52]. Besides, as a result of the DRUP design, it should be noted that these results were obtained in a small sample size and therefore require validation in a larger cohort. Nevertheless, we detected a clinically relevant signal of activity of durvalumab across various advanced dMMR/MSI-H solid tumours and it thus shows that studies like DRUP can contribute significantly to the identification of clinical signs of activity.

## Conclusion

In conclusion, the PD-L1 inhibitor durvalumab provided durable responses in previously treated patients with advanced dMMR/MSI-H solid tumours with CB in 50% and an OR in 27%. Biomarker analyses revealed high SV burden, *JAK1* frameshift mutations and low IFN-γ expression as possible resistance mechanisms to anti-PDL1 in dMMR/MSI-H tumours, providing a rationale for larger studies to validate these findings.

## Supplementary Information

**Additional file 1.**

**Additional file 2.**

## Data Availability

All data described in this study are freely available for academic use, and can be obtained through the Netherlands Cancer Institute and the HMF through standardized procedures and request forms. These can be found at https://www.hartwigmedicalfoundation.nl/en. For further information, see van der Velden (Nature, 2019).

## Abbreviations

AEs: Adverse events
CB: Clinical benefit
CI: Confidence Interval
CR: Complete response
CRC: Colorectal cancer
CTCAE: Common Terminology Criteria for Adverse Events
dMMR: Mismatch repair deficient
DoR: Duration of response
DRUP: Drug Rediscovery Protocol
ICI: Immune checkpoint inhibitors
IFN-γ: IFN-gamma
IHC: Immunohistochemistry
indels: insertions and deletions
Mb: Megabase
ML: Tumour mutational load
MSI(-H): Microsatellite instability(-high)
MSS: Microsatellite stable
msIndels: Microsatellite-instable indels
NGS: Next generation sequencing
ORR: Objective response rate
OS: Overall survival
PCR: Polymerase chain reaction
PD-1: Programmed cell death 1
PD-L1: Programmed cell death ligand 1
PFS: Progression free survival
PR: Partial response
RANO: Response Assessment in Neuro-Oncology
RECIST: Response Evaluation Criteria in Solid Tumours version 1.1
SD: Stable disease
SV: Structural variant
TCGA: The Cancer Genome Atlas
TMB: Tumour mutational burden
WGS: Whole genome sequencing
