# Supplementary material for: Efficacy, safety and biomarker analysis of durvalumab in patients with mismatch-repair deficient or microsatellite instability-high solid tumours
Source: BMC Cancer. 2023 Mar 4;23:205. doi: 10.1186/s12885-023-10663-2 (PMC9985217; doi:10.1186/s12885-023-10663-2)
Supplement: Supplementary file 2 — Additional file 2. [file 12885_2023_10663_MOESM2_ESM.pdf]

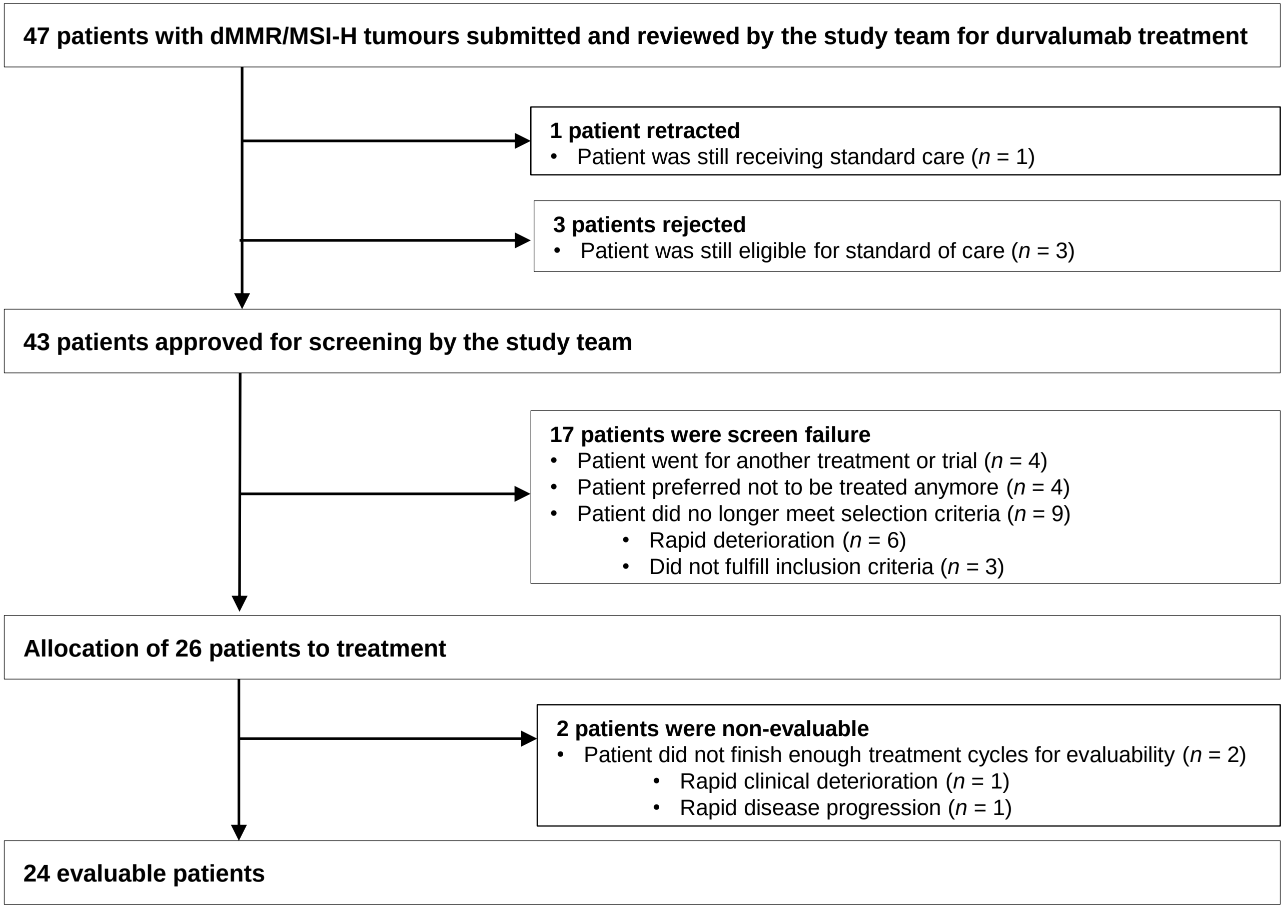

Supplementary Figure S1: Flowchart

Flowchart of patients submitted to the study between January 2019 and April 2020, and reasons for rejection, screen failure and non-evaluability.
